# Individual and community-level determinants of knowledge of ovulatory cycle among women of reproductive age in 29 African countries: a multilevel analysis

**DOI:** 10.1186/s12905-022-01984-8

**Published:** 2022-09-29

**Authors:** Betregiorgis Zegeye, Nicholas Kofi Adjei, Dina Idriss-Wheeler, Sanni Yaya

**Affiliations:** 1 HaSET Maternal and Child Health Research Program, Shewarobit Field Office, Shewarobit, Ethiopia; 2 Department of Public Health, Policy and Systems, University of Liverpool, Liverpool, UK; 3 Interdisciplinary School of Health Sciences, University of Ottawa, Ottawa, Canada; 4 School of International Development and Global Studies, Faculty of Social Sciences, University of Ottawa, 120 University Private, Ottawa, ON K1N 6N5 Canada; 5 The George Institute for Global Health, Imperial College London, London, UK

**Keywords:** Knowledge of ovulatory cycle, Fertility, Women's health, Africa, Demographic Health Survey (DHS), Global health

## Abstract

**Background:**

Knowledge of the ovulatory cycle (KOC) can help reduce the chances of unwanted pregnancies and may improve a woman’s reproductive health. However, little is known about the factors associated with knowledge of the ovulatory cycle across Africa. Therefore, we aimed to investigate the individual/household and community level determinants of KOC among women of childbearing age in 29 African countries.

**Methods:**

We used data from the Demographic and Health Surveys of 29 African countries conducted between 2010 and 2020. Bivariate and multivariate multilevel logistic regressions were used to examine the association between women’s correct knowledge of the ovulatory cycle and individual/household and community-level factors. The results were reported using adjusted odds ratios (AOR) with a 95% confidence interval (CI).

**Results:**

The pooled results showed that correct KOC among women was 15.5% (95% CI 14.2–17.0%), varying from 11.5% in Liberia to 57.1% in the Democratic Republic of Congo. Regarding regional distribution, the highest prevalence of KOC was observed in West Africa (38.8%) followed by East Africa (21.3%) and was lowest in Southern Africa (15.6%) and Central Africa (15.5%). After adjusting for potential confounders, at the individual level, we found the odds of KOC to be higher among older women (40–44 years-aOR 3.57, 95% CI 1.90–6.67, 45–49 years-aOR 2.49, 95% CI 1.29–4.82), and women with higher educational level (aOR 2.58, 95% CI 1.40–4.75); at the community level, higher KOC was among women exposed to media (aOR 2.24, 95% CI 1.32–3.81).

**Conclusions:**

Knowledge of ovulatory cycle among women of reproductive age was found to be low in the region and varied by country. Women’s age and educational level were the individual-level factors associated with increased knowledge of ovulatory cycle while community-level media exposure was found to be associated with increased knowledge of ovulatory cycle in this study. This finding highlights the need for appropriate strategies (possibly use of mass media) to increase knowledge of ovulatory cycle among women of reproductive age, especially among adolescents in Africa.

## Background

The term ovulation refers to the process when the mature egg is released from the ovary to the fallopian tube in preparation for fertilization [1]. Key physiological events indicate ovulation is taking place such as changes in basal body temperature (BBT) and presence of cervical mucus [2]. Natural family planning methods require knowledge of the ovulation cycle along with other approaches such as BBT, presence of cervical mucus and length of menstrual cycle.

Though it requires more knowledge, the natural family planning strategy such as Billings Ovulation Method (BOM) can be successful to help plan or avoid pregnancy. BOM is based on observing mucus patterns in the menstrual cycle which signal proximity to ovulation. Cervical secretions change close to ovulation, and with proper knowledge and guidance, women can recognize this sign of fertility. Subsequently, and in agreement with their partner, they choose to have sex or abstain from it, according to the desire to conceive or not [3, 4].

Therefore, knowledge of the ovulatory cycle (KOC) can be an effective family planning method to make decisions about pregnancy and fertility [5, 6] Additionally, knowing and understanding ovulation can help diagnose certain pathologies or medical conditions while also decreasing chances of unintended pregnancy [7].

Unintended pregnancy is either unwanted or unplanned for at least one of the couples [8, 9]. Usually, an unplanned pregnancy is mistimed and the child is wanted. The unwanted pregnancy has become a major public health and reproductive health concern [8], with negative consequences for the mother, baby, and the general public [10]. Mothers with unwanted pregnancies may be exposed to several devastating complications such as induced abortion leading to maternal death, higher crime rates among birth cohorts, maternal depression, and family stress, reduced employment effectiveness and reduced school performance [10]. For adolescent girls in LMICs, complex and interrelated outcomes of child marriage, dropping out of school and early pregnancies are associated with gender inequities, illiteracy, single motherhood, unemployment and other negative social outcomes [11]. Unintended pregnancy is also associated with an array of negative outcomes for the women including less stable romantic relationships, and higher incidence of mental-health problems associated with delinquent behaviour during teenage years for the child [12].

In an era of increasing health risks, denial, discontinuation, and a high unmet need for modern contraception, knowledge about the timing of ovulation in reproductive women is necessary [13]. The fear of side effects of modern contraception was the most reported reason for non-use of contraception and modern contraception in particular. A study in Nigeria documented that some women believe that modern contraceptives are harmful to the body and as such fail to use them or rely on less effective traditional family planning methods without the necessary knowledge [14].

Knowledge of the ovulation cycle (KOC) is essential for successful practice of intercourse-related methods such as periodic abstinence, abstinence, and condom use [15], especially when sexually active young adults may have limited access to modern contraceptive methods [16]. Nonetheless, effective periodic abstinence may depend on the man's knowledge of the method [17], as well as cooperation during the ovulation period [15]. Ultimately, effective periodic abstinence depends not only on the man’s knowledge, but also the woman’s knowledge and man’s cooperation [4, 18].

Even in high-income countries, KOC is low [14]. For example, in the United States, approximately 32.8% of women have correct knowledge of the ovulatory cycle [16]. A study in Spain showed the prevalence is around 31.2% [19]. A study in India also showed low prevalence of KOC (15%) [20]. A previous estimate in Ethiopia revealed that only one in four women know their most fertile period [13, 21]. Another study in Togo and Ghana showed that knowledge of the ovulatory cycle was 42.8% [22] and 38%, [23] respectively.

Previous studies have identified socioeconomic, demographic, unwanted pregnancies, and media exposure factors to be associated with KOC in some developing countries [13, 16, 19–23]. Although there is a study that attempted to assess the relationship between these factors and KOC in some African countries [22], community-level factors have yet to be explored. Investigating community-level factors may help to develop appropriate health strategies and interventions [21]. Therefore, this study aimed to identify KOC factors at the individual/household and community level among women of reproductive age in 29 African countries.

## Methods

### Data source

Demographic and Health Surveys (DHSs) of 29 African countries, conducted between 2010 and 2020, were pooled and used in this study. The surveys were nationally representative of men and women aged between 15 and 49 years. The surveys include data on a wide range of public health-related issues including demographic characteristics, socioeconomic status (SES), anthropometric measures, maternity history, family planning and domestic violence and knowledge of ovulatory cycle [24]. Details of the sampling procedure and data collection methods are outlined in the Guide to DHS Statistics [25]. Studied countries are selected based on the criteria of availability of outcome variable and key explanatory variables, and with DHS surveys conducted between 2010 and 2020. A total of 383,131 women of reproductive age were used for the analysis. The DHS datasets are available in the public domain and can be accessed at http://dhsprogram.com/data/available-datasets.cfm. Table 1 provides detailed information about selected countries, year of survey, and samples.

**Table 1:** Survey year, included country and their respective sampled population

Country	Year	Sampled population
Angola	2015/16	14,379
Burkina Faso	2010	17,087
Benin	2017/18	15,928
Burundi	2016/17	17,269
Congo	2013/14	18,802
Democratic Republic of Congo	2011/12	10,819
Côte d’Ivoire	2011/12	10,047
Cameroon	2018/19	13,527
Ethiopia	2016	15,683
Gabon	2012	8422
Ghana	2014	9395
Gambia	2013	10,172
Guinea	2018	10,874
Kenya	2014	14,724
Comoros	2012	5329
Liberia	2019/20	8065
Mali	2018	10,519
Malawi	2015/16	24,562
Niger	2012	11,160
Namibia	2013	9166
Rwanda	2014	13,484
Sierra Leone	2019	15,574
Senegal	2010/11	15,688
Chad	2014/15	17,580
Togo	2013/14	9468
Tanzania	2015/16	13,264
Uganda	2016	18,506
Zambia	2018/19	13,683
Zimbabwe	2015	9955
Total		383,131

### Study variables

#### Outcome variable

The outcome variable for this study was women’s correct knowledge of ovulatory cycle (KOC). In the DHS, the question on KOC answered by women of reproductive age was "when is the ovulation time?". Response options were: “during her period", "after period ended", "middle of the cycle", "before the period begins", "at any time", and "don’t know". The outcomes variable was recoded and all respondents who indicated “middle of the cycle” which were considered as correct knowledge of ovulatory cycle and coded as “1”, and the other responses, incorrect knowledge of ovulatory cycle, were coded as “0” [21, 26, 27]. Specifically, this study’s methodology follows the work completed by Dagnew et al. [21] which looked at individual and community-level determinants of knowledge of ovulatory cycle among women of childbearing age in Ethiopia [21].

#### Explanatory variables

Based on evidence from previous studies [13, 16, 19–23], individual/household level and community-level explanatory variables were considered for this current study and pooled from the 29 countries listed in Table 1.

#### Individual/household level explanatory variables

The individual/household level explanatory factors included women’s age in years (15–19, 20–24, 25–29, 30–34, 35–39, 40–44, 45–49), women’s educational level (no formal education, primary school, secondary school, higher), husband’s educational level (no formal education, primary school, secondary school, higher), marital status (not married, married), currently employed (no, yes) and parity (0, 1–2, 3–4, 5+). Exposure to media [(newspaper, radio, or television (TV)] was assessed in terms of frequency (no exposure, less than once a week, at least once a week, at least less than once and a week) and wealth index (poorest, poorer middle, richer and richest) [28, 29].

#### Community-level explanatory variables

The community-level factors were distance to health facility (big problem, not a big problem), place of residence (urban, rural), community literacy level (low, medium, high), community poverty level (low, medium, high) and community media exposure level (low, medium, high). Community-level variables were generated by aggregating the individual level data into a cluster except for place of residence and distance to health facility that were already community level variables. In DHS, place of residence was one of the characteristics that helped in designing the sample to give population and health indicators at the national level. The other community-level variables were obtained by aggregating the individual women characteristics into clusters. They were computed using the proportion of a given variables’ subcategory per cluster. Since the aggregate values for all generated variables have no meaning at the individual level, they were categorized into groups. The occupation, education, and wealth of survey participants in each community were used to compute community-level SES. Principal component analysis (PCA) was used to calculate women who were unemployed, uneducated, and poor. A standardized score was derived, with a mean score (0) and standard deviation (1). These were then categorized into tertile 1 (lowest score, least disadvantaged and greater SES), tertile 2 and tertile 3 (highest score, most disadvantaged and lowest SES). To determine the community literacy level, respondents who attended higher than secondary school were assumed to be literate, while all other respondents were given a sentence to read and were considered literate if they could read all or part of the sentence. As a result, respondents who had completed at least a secondary education or who had completed just elementary or primary school but could read a complete sentence were considered to have high literacy. Respondents with medium literacy could read portions of sentences and did not attend school or have a primary or secondary education. Respondents with low literacy were those who had never attended school or received only elementary or secondary education. These were divided into appropriate tertiles, with tertile 1 (lowest score, least disadvantaged) representing strong community literacy, tertile 2 (middle score), medium community literacy, and tertile 3 (highest score, most disadvantaged), representing low community literacy. Community-level media exposure refers to the percentage of women who, in the cluster, had at least some exposure to radio, television, or newspapers. The same process as described above was used to develop a variable for community media exposure [30, 31].

### Statistical analyses

Descriptive analysis was performed using frequency and percentage distributions to examine the characteristics of respondents and knowledge of ovulatory cycle. This was followed by bivariate multilevel logistic regression to select variables that had a significant association with knowledge of ovulatory cycle at p-value less than 0.5. A multicollinearity test was performed using variance inflation factor (VIF) for all statistically significant variables at the bivariate multilevel logistic regression. We found no evidence of collinearity among the explanatory variables (Mean VIF = 1.69, Min VIF = 1.04, Max VIF = 2.77). Using multilevel logistic regression (MLLR) method, we created four different models to assess whether the individual/household and community-level factors had significant associations with the outcome variable (knowledge of ovulatory cycle). The first model was a null model (Model 0), which had no explanatory variables, and it showed variance in knowledge of ovulatory cycle, attributed to primary sampling Unit (PSU). The second model (model I) comprised individual/household-level factors and the third model (Model II) comprised community-level factors. The last model, (Model III), was the complete model that included factors at both the individual/household and community levels.

All four MLLR models included fixed and random effects [32, 33]. The fixed-effects model showed the association between the explanatory variables and the outcome variable, and the random effects signified the measure of variation in the outcome variable based on PSU, which was measured by Intra-Cluster Correlation (ICC) [34]. The model fit was assessed using the Akaike’s Information Criterion (AIC) [35]. We used the “mlogit” command to run the MLLR models. The “svyset” command was used to adjust for survey weight, cluster, and strata. The analyses were performed using Stata version-14 software (Stata Corp, College Station, Texas, USA). We also followed the guidelines for Strengthening Observational studies in Epidemiology (STROBE) [36].

### Ethical clearance

Publicly available secondary data was used in this study (available at: https://dhsprogram.com/data/available-datasets.cfm). Ethical procedures were completed by the institutions that funded, commissioned, and managed the surveys, and no further ethical clearance was required. ICF international ensured that all the DHS surveys follow the U.S. Department of Health and Human Services rules for the respect of human subjects’ rights. More details about data and ethical standards are available http://goo.gl/ny8T6X.

## Results

### Background characteristics of respondents

A total of 383,131 women of reproductive age (15–19 years) were included in the analyses. About 24.0% and 21.2% of the women were in the (15–19 years) and (20–24 years) age group, respectively. Approximately 22.1% of the respondents and 13.4% of their husbands had no formal education. Around 30.4% of respondents were rural residents and 26.2% were not exposed to media. More than half (51.8%) of the respondents had a big problem reaching a health facility (Table 2).

**Table 2:** Knowledge of ovulatory cycle among women of reproductive age in Africa sub regions: evidence from DHS of 29 countries

Sub region	Included country	Prevalence of ovulatory knowledge [Estimate [95% CI]]
Central Africa	Angola	15.5% [14.1–17.0%]
	Congo	
	Democratic Republic of Congo	
	Cameroon	
	Gabon	
	Chad	
East Africa	Burundi	21.3% [20.1–22.6%]
	Ethiopia	
	Kenya	
	Comoro	
	Malawi	
	Rwanda	
	Tanzania	
	Uganda	
	Zambia	
	Zimbabwe	
Southern Africa	Namibia	15.6% [14.5–16.8%]
West Africa	Burkina Faso	38.8% [37.0–40.7%]
	Benin	
	Côte d’Ivoire	
	Ghana	
	Gambia	
	Guinea	
	Liberia	
	Mali	
	Niger	
	Sierra Leone	
	Senegal	
	Togo	

### Prevalence of knowledge of ovulatory cycle across countries

**Fig. 1:**
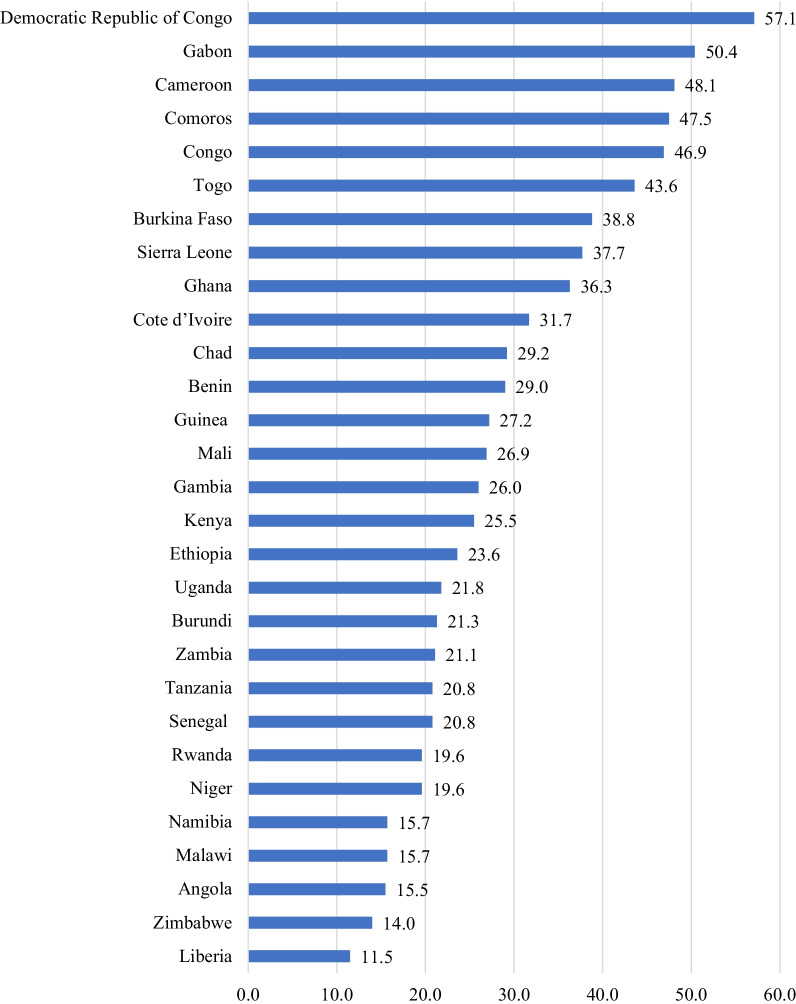
Knowledge of ovulatory cycle among women of reproductive age in SSA: evidence from DHS of 29 countries (N = 383,131)

The prevalence of knowledge of ovulatory cycle among women of reproductive age in the 29 sampled African countries was 15.5%. The highest prevalence was observed in the Democratic Republic of Congo (57.1%), Gabon (50.4%) and Cameroon (48.1%). We observed the lowest prevalence of knowledge of ovulatory cycle in Liberia (11.5%), Zimbabwe (14.0%) and Angola (15.5%) (Fig. 1).

Regarding regional distribution, the highest prevalence of knowledge of ovulatory cycle was found in West Africa (38.8%) followed by East Africa (21.3%), Southern Africa (15.6%) and Central Africa (15.5%) (Table 2).

### Prevalence of knowledge of ovulatory cycle across explanatory variables

Table 3 shows the distribution of knowledge of ovulatory cycle by explanatory variables and subgroups. We observed that only 8.6% of women who had no formal education had correct knowledge of ovulatory cycle. Knowledge of ovulatory cycle varied from 7.8% among women in the poorest household to 25.3% among women in the richest household category. Differences in knowledge of ovulatory cycle also varied by residence, with a prevalence of 18.4% in the urban areas and 8.9% in the rural areas. We further observed a prevalence of 7.0% among women from low community media exposure and 21.9% among women from high-level media exposure community (Table 3).

**Table 3:** Knowledge of ovulatory cycle among women of reproductive age across explanatory variables in Africa: evidence from DHS of 29 countries (N = 383,131)

Variable	Frequency (Weighted %)	Knowledge of ovulatory cycle	COR [95% CI]
*Women’s age in years*			
15–19	82,603 (24.0)	12.0	Ref
20–24	71,379 (21.2)	15.4	1.47 (1.16–1.87)**
25–29	66,564 (17.1)	17.5	1.71 (1.33–2.20)***
30–34	54,298 (12.4)	18.4	1.71 (1.29–2.28)***
35–39	46,200 (10.5)	14.7	1.44 (1.10–1.89)**
40–44	34,482 (8.6)	18.5	1.79 (1.32–2.43)***
45–49	27,605 (6.2)	15.8	1.65 (1.24–2.20)**
*Women’s educational level*			
No formal education	130,603 (22.1)	8.58	Ref
Primary school	124,382 (34.8)	12.0	1.28 (1.02–1.62)*
Secondary school	113,639 (38.3)	20.1	1.98 (1.56–2.52)***
Higher	14,463 (4.8)	36.7	3.51 (2.25–5.46)***
*Husband’s educational level*			
No formal education	94,297 (13.4)	8.1	Ref
Primary school	68,536 (29.2)	11.5	1.35 (0.97–1.88)
Secondary school	69,989 (49.3)	18.8	1.99 (1.44–2.76)***
Higher	17,328 (8.1)	26.7	2.27 (1.42–3.64)**
*Economic status*			
Poorest	78,290 (16.9)	7.8	Ref
Poorer	73,319 (17.6)	9.2	1.31 (1.01–1.70)*
Middle	72,742 (19.5)	12.3	1.68 (1.29–2.19)***
Richer	74,117 (22.5)	18.8	2.82 (2.14–3.72)***
Richest	84,663 (23.6)	25.3	3.25 (2.43–4.34)***
*Place of residence*			
Urban	141,992 (69.6)	18.4	Ref
Rural	241,139 (30.4)	8.9	0.51 (0.41–0.64)***
*Media exposure*			
No	124,486 (26.2)	7.9	Ref
Yes	257,938 (73.8)	18.2	1.68 (1.37–2.07)***
*Distance to health facility*			
Big problem	146,746 (51.8)	13.7	Ref
Not a big problem	224,733 (48.2)	17.5	1.19 (0.97–1.46)
*Marital status*			
Not married	186,893 (89.2)	15.2	Ref
Married	196,238 (10.8)	18.0	1.19 (0.94–1.50)
*Currently employed*			
No	155,544 (34.9)	13.9	Ref
Yes	227,017 (65.1)	16.4	1.41 (1.16–1.70)***
*Parity*			
0	102,282 (24.9)	15.2	Ref
1–2	53,610 (15.1)	18.6	1.49 (1.17–1.90)**
3–4	129,304 (34.5)	15.3	1.24 (1.01–1.53)*
5+	97,935 (25.5)	14.4	1.31 (1.06–1.63)*
*Community literacy level*			
Low	130,514 (24.9)	8.2	Ref
Medium	125,832 (32.2)	11.1	1.41 (1.08–1.84)*
High	126,785 (43.0)	23.1	3.35 (2.62–4.28)***
*Community level contraceptive knowledge*			
Low	249,755 (27.0)	8.5	Ref
Medium	92,924 (30.4)	14.8	1.71 (1.31–2.22)***
High	40,452 (42.6)	20.5	2.50 (1.91–3.27)***
*Community level poverty*			
Low	132,315 (49.1)	21.4	Ref
Medium	128,993 (23.7)	11.8	0.50 (0.39–0.64)***
High	121,823 (27.1)	8.2	0.34 (0.26–0.44)***
*Community level media exposure*			
Low	134,267 (25.9)	7.0	Ref
Medium	139,448 (25.4)	11.9	1.79 (1.37–2.33)***
High	109,416 (48.7)	21.9	3.52 (2.72–4.55)***

***p<0.001, **p<0.01, *p<0.05

### Fixed effect (measure of association)

Table 4 shows the fixed effects results of the individual and community level factors associated with knowledge of ovulatory cycle.

**Table 4:** Multilevel multivariable logistic regression results of knowledge of ovulatory cycle and its individual/household and community level factors in Africa: evidence from DHS of 29 countries (N = 383,131)

Variable	Model I AOR [95% CI]	Model II AOR [95% CI]	Model III AOR [95% CI]
*Women’s age in years*			
15–19 (Ref)			
20–24	1.72 (1.00–2.96)*		1.70 (0.98–2.92)
25–29	2.29 (1.28–4.08)**		2.22 (1.25–3.95)**
30–34	2.97 (1.62–5.44)***		2.84 (1.55–5.21)**
35–39	2.40 (1.28–4.49)**		2.30 (1.23–4.31)**
40–44	3.74 (1.99–7.03)***		3.57 (1.90–6.67)***
45–49	2.59 (1.34–5.01)**		2.49 (1.29–4.82)**
*Women’s educational level*			
No formal education (Ref)			
Primary school	1.24 (0.91–1.69)		1.23 (0.90–1.68)
Secondary school	1.62 (1.14–2.31)**		1.57 (1.09–2.26)*
Higher	2.72 (1.47–5.02)**		2.58 (1.40–4.75)**
*Husband’s educational level*			
No formal education (Ref)			
Primary school	1.13 (0.79–1.60)		1.08 (0.76–1.54)
Secondary school	1.34 (0.92–1.96)		1.28 (0.87–1.87)
Higher	1.07 (0.61–1.86)		1.01 (0.58–1.76)
*Economic status*			
Poorest (Ref)			
Poor	1.16 (0.85–1.60)		1.14 (0.81–1.60)
Middle	1.30 (0.88–1.91)		1.12 (0.69–1.83)
Rich	1.70 (1.123–2.59)*		1.29 (0.71–2.34)
Richest	1.65 (1.05–2.60)*		1.20 (0.64–2.26)
*Media exposure*			
No (Ref)			
Yes	1.19 (0.88–1.61)		1.08 (0.80–1.47)
*Currently employed*			
No (Ref)			
Yes	1.08 (0.81–1.44)		1.08 (0.81–1.44)
*Parity*			
0 (Ref)			
1–2	1.23 (0.69–2.18)		1.23 (0.69–2.18)
3–4	0.86 (0.48–1.54)		0.87 (0.49–1.56)
5+	0.94 (0.53–1.68)		0.97 (0.54–1.74)
*Distance to health facility*			
Big problem (Ref)			
Not a big problem		1.12 (0.91–1.37)	1.02 (0.79–1.31)
*Place of residence*			
Urban (Ref)			
Rural		1.36 (0.91–2.04)	1.41 (0.89–2.25)
*Community literacy level*			
Low (Ref)			
Medium		1.15 (0.83–1.60)	0.93 (0.64–1.33)
High		2.08 (1.42–3.05)***	1.36 (0.86–2.13)
*Community level contraceptive knowledge*			
Low (Ref)			
Medium		1.09 (0.81–1.46)	1.09 (0.78–1.52)
High		1.01 (0.71–1.44)	1.02 (0.68–1.52)
*Community level poverty*			
Low (Ref)			
Medium		0.92 (0.67–1.25)	1.02 (0.69–1.50)
High		1.00 (0.58–1.73)	1.10 (0.53–2.27)
*Community level media exposure*			
Low (Ref)			
Medium		1.79 (1.22–2.61)**	1.55 (0.99–2.44)
High		2.53 (1.61–3.96)***	2.24 (1.32–3.81)**

***p<0.001, **p<0.01, *p<0.05

#### Individual/household level factors

The results showed that the likelihood of knowledge of ovulatory cycle among women within the age groups of 25–29 years (aOR 2.22, 95% CI 1.25–3.95), 30–34 years (aOR 2.84, 95% CI 1.55–5.21), 35–39 years (aOR 2.30, 95% CI 1.23–4.31), 40–44 years (aOR 3.57, 95% CI 1.90–6.67), 45–49 years (aOR 2.49, 95% CI 1.29–4.82) were higher compared to women within the age groups of 15–19 years. Similarly, higher odds of knowledge of ovulatory cycle was observed among women who completed secondary school (aOR 1.57, 95% CI 1.09–2.26) and higher (aOR 2.58, 95% CI 1.40–4.75) compared to those with no formal education.

#### Community-level factors

Regarding community-level factors, we found higher odds of knowledge of ovulatory cycle among women from high community-level media exposure (aOR 2.24, 95% CI 1.32–3.81) compared to those from low community-level media exposure (Table 4).

### Random effects (measures of variations) results

The random effect models of the individual/household and community level factors associated with knowledge of ovulatory cycle are shown in Table 5. We observed that the values of the AIC decreased across the models, indicating a best-fitted model. The ICC in the null model (ICC = 0.30) showed that the odds knowledge of ovulatory cycle varied across clusters (σ2 = 1.04, 0.85–1.29). The between-cluster variations decreased by 8% in model I, from 30% in the null model to 22% in model I. From model I, the ICC increased again by 3% Model II (ICC = 0.25) and then declined by 4% in the complete model (Model III, ICC = 0.21. These estimates showed that the variations in the likelihood of knowledge of ovulatory cycle can be attributed to the variances in the clustering at the primary sampling units (Table 5).

**Table 5:** Random effect results for knowledge of ovulatory cycle and its individual and community level factors: evidence from DHS of 29 countries (N = 383,131)

Random effect	Model 0	Model I	Model II	Model III
PSU variance (95% CI)	1.04 (0.85–1.29)	0.70 (0.51–0.96)	0.68 (0.52–0.90)	0.63 (0.45–0.89)
ICC	0.30	0.22	0.25	0.21
LR Test	764.12	166.36	521.17	147.96
Wald chi-square and p-value	Ref	χ2 = 123.32, *p* < 0.001	χ2 = 129.21, *p* < 0.001	χ2 = 155.34, *p* < 0.001
*Model fitness*				
Log-likelihood	− 5749.46	− 2878.09	− 5688.83	− 2866.26
AIC	11,502.92	5802.18	11,401.67	5798.53
N				

Ref, reference category; AIC, Akaike Information Criterion; PSU, Primary Sampling Unit; N, total observation; LR, Likelihood Ratio; ICC, Intra-class correlation coefficient

## Discussion

In this study, we investigated knowledge of ovulatory cycle and its individual/household and community level factors among women of reproductive age using nationally representative datasets from 29 African countries. Overall, the pooled results showed that approximately 15.5% (95% CI 14.2%-17.0%) of women of reproductive age had correct knowledge of ovulatory cycle. A study by Iyanda et al. [22] in Africa, looking specifically at 15–24-year-old women, showed that 26% of 15–26-year-olds had correct knowledge of ovulation [22]. Our study, when broken down by age group, shows similar findings amongst the younger cohort of women (27% of 15–24-year-olds had correct KOC).

Our findings showed that women’s age was associated with knowledge of ovulatory cycle, where older women were more likely to have correct knowledge of ovulatory cycle than teenagers; this is consistent with prior findings in Ethiopia and Ghana [13, 21, 23]. A reason for this outcome could be that as age increases, exposure to different reproductive-related issues also increases, enhancing women’s sexual and reproductive knowledge [21].

We found that women’s educational level was associated with correct knowledge of ovulatory cycle; similar to findings in Uganda [37], Ethiopia [15] and a systematic review conducted by Pedro et al. [38]. As Getahun & Nigatu (2020) suggest, this association may be due to likelihood of increased knowledge of the physiology of reproduction by women with higher education [13]. Several countries have created educational interventions to increase awareness of fertility [39]. Success in increased fertility awareness after exposure to a fertility education website was found in a Canadian context [39]. In Rwanda and Spain, an entertainment-based radio drama and a randomized control trial featuring oral education, respectively, increased reproductive-aged women’s awareness of fertility [19, 40]. Additionally, increased fertility awareness and education have been positively associated with increased family planning utilization [41, 42].

Interestingly, individual media exposure was not significantly associated with correct knowledge of ovulatory cycle, however, high community-level media exposure was significantly associated KOC; the latter finding is consistent with prior work in Ethiopia [21]. It did not matter if there was no exposure versus some exposure, whereas women in communities with high media-exposure were more likely to have corrected KOC. Studies have shown that communities influence family planning (FP) utilization (including traditional methods) through prevailing fertility norms, gender disparities, health knowledge, social networks, community health worker (CHW) programs and mass communication exposure to family planning messages within media [43–45]. A systematic review by Scott et al. (2015) revealed that 83 percent of included studies reported an improvement in contraceptive knowledge and attitudes resulting from CHW FP programs [46]. In a recent 2021 study in the Philippines and Myanmar, mass media has been suggested as an effective tool for influencing knowledge of ovulation and contraceptive use as well as promoting health-related behaviours (i.e., reproductive preferences) [47]. A study in Africa pooled findings from 47 countries and revealed 44% of women were exposed to mass media related to family planning  [48]. Community-level media exposure has been shown to increase maternal health service utilization  [49], which can be seen as a way of disseminating health information regarding family planning such as knowledge of the reproductive cycle [21, 49]. Of note, a study in Nigeria on access to mass media and use of family planning found that people with higher socio-economic status had more access to mass media, especially television and radio, than people with lower socio-economic status  [50, 51]. There was no significant finding between wealth at the individual level or community level poverty and correct KOC in our study, whereas other studies have found significance between individual wealth status and knowledge of ovulatory cycle [21, 22]. Although findings are mixed in terms of socioeconomic, wealth and community-level poverty and correlation with education and access to mass media across Africa, there is evidence for potential use of this method to disseminate correct KOC and other sexual and reproductive health or maternal health education information.

### Strengths and limitations of the study

A key strength in this study is the use of pooled data (i.e., enhanced statistical power) from nationally representative data sets across 29 countries in Africa to compare outcomes across countries and specific sub-regions. Additionally, we were able to study correct knowledge of the ovulatory cycle by looking at two levels—individual/household and community—which allowed us to study hierarchical or clustered structures that may influence outcomes. Our study has some limitations. Due to the cross-sectional nature of the study, we can only use predictive modeling to determine associations with no ability to determine any causal-effect relationships. The DHS relies on self-reported data and is subject to recall bias. Furthermore, we must acknowledge that the pooled data may have included the same question but were from different time points (2010–2020) in the selected countries.

## Conclusion

Knowledge of ovulatory cycle among reproductive-aged women was found to be low in the 29 African countries in this study. Women with advanced age and those who had formal education were the significant individual/household level factors associated with increased knowledge of ovulatory cycle while community level media exposure was found to be the only identified community-level factor associated with increased knowledge of ovulatory cycle in our study. In African countries where modern contraceptive method utilization is not sufficient, the low prevalence of knowledge of ovulatory cycle, a more traditional and accepted family planning method, is concerning. Our findings highlight the need for appropriate strategies to increase correct knowledge of ovulatory cycle among women of reproductive age, especially adolescents in Africa. Implications for increasing fertility-related knowledge and behaviours in the region should be considered and transmitted through mass media information, education, and communication.

## Data Availability

Data used in this study were obtained from the DHS Program and available at: https://dhsprogram.com/data/available-datasets.cfm.

## Abbreviations

KOC: Knowledge of the ovulatory cycle
DHS: Demographic and Health Surveys
CI: Confidence Interval
